# Machine learning analysis based on deep learning for fatigue diagnostics in carbon fiber reinforced polymers

**DOI:** 10.1371/journal.pone.0340904

**Published:** 2026-01-09

**Authors:** Ahmed Salah Al-Shati, Thamer J. Mohammed

**Affiliations:** Department of Chemical Engineering Processes, College of Chemical Engineering, University of Technology, Baghdad, Iraq; Zhejiang University, CHINA

## Abstract

Fatigue-induced degradation in Carbon Fiber Reinforced Polymer (CFRP) structures poses a critical challenge in long-term structural health monitoring (SHM) applications. In this study, a hybrid deep learning framework is proposed for fatigue state classification of CFRP composites using sensor-based monitoring data. The framework integrates a one-dimensional Convolutional Neural Network (1D-CNN) to extract spatial degradation patterns and an extended Long Short-Term Memory (xLSTM) network to capture long-range temporal dependencies associated with fatigue evolution. The extracted spatiotemporal features are fused and refined through Mutual Information-based feature selection, followed by a Bagging-based ensemble classifier for robust fatigue state discrimination. The proposed approach is evaluated using the NASA-CFRP dataset, achieving an average classification accuracy of 99%. While the framework is generally applicable to SHM of CFRP structures, its relevance to membrane-based gas separation systems is discussed as a representative application scenario. The results demonstrate the effectiveness of the proposed method for reliable fatigue diagnosis and maintenance decision support in CFRP-based engineering systems.

## 1. Introduction

Carbon Fiber Reinforced Polymer (CFRP) composites are widely used in aerospace, energy, and industrial systems due to their high strength-to-weight ratio, corrosion resistance, and excellent fatigue performance [1]. Despite these advantages, CFRP structures are inherently susceptible to fatigue damage under cyclic mechanical and environmental loading, making early fatigue diagnosis a critical requirement in Structural Health Monitoring (SHM) applications [2]. Reliable detection of fatigue states enables condition-based maintenance, improves operational safety, and reduces lifecycle costs in CFRP-based systems.

In certain industrial applications, such as membrane-based gas separation systems, CFRP components are subjected to cyclic pressure loading, temperature variations, and prolonged exposure to aggressive gaseous environments, particularly hydrogen-rich conditions [3]. These operating conditions introduce unique degradation mechanisms that significantly increase the importance of fatigue monitoring. Hydrogen, due to its small molecular size and high diffusivity, can penetrate polymeric and composite materials, potentially leading to hydrogen-induced material degradation, permeability variation, and microstructural damage [4].

Despite the mechanical advantages of CFRPs, fatigue-related damage such as microcracking, matrix weakening, and delamination remains a critical concern, especially in long-term industrial applications. Early detection of fatigue is essential to optimize filter replacement schedules and reduce maintenance costs. In this context, Structural Health Monitoring (SHM) and Prognostics and Health Management (PHM) techniques have emerged as valuable tools for assessing the condition of composite structures [5,6]. Several SHM methods—including acoustic emission, ultrasonic testing, and strain monitoring—have been adapted to membrane-based systems. However, traditional approaches often lack automation and fail to capture subtle temporal degradation patterns [7].

Recent advances in data-driven modeling have enabled more accurate and scalable diagnostics. Traditional machine learning methods such as Support Vector Machines (SVMs), Random Forests, and k-Nearest Neighbors have shown potential in detecting faults in composite structures [8]. More recently, deep learning architectures such as CNNs and LSTMs have been introduced for SHM applications [9,10]. CNNs are effective in capturing local patterns, while LSTMs model temporal dependencies. However, few studies have explored their integration in the context of fatigue detection for polymeric membranes [11].

The NASA-CFRP dataset is one of the highly valuable sources within this domain that provides actual operation information of the fatigue behavior of CFRP materials under different loading conditions. Nonetheless, the dataset has several difficulties: unbalanced class distribution between healthy and fatigued samples, measurement noise, as well as the complex spatial-temporal structure of the data [12]. Previous approaches have struggled to simultaneously address these challenges while effectively capturing both spatial and temporal information.

In this study, we propose a hybrid CNN-xLSTM deep learning framework tailored for fatigue diagnostics in CFRP membranes. The CNN captures spatial patterns in the raw sensor signals, while the xLSTM models temporal evolution of fatigue. The extracted features are fused and refined using Mutual Information-based feature selection, and a Bagging-based ensemble classifier is used to distinguish between healthy and fatigued states. This hybrid approach not only fully leverages both spatial and temporal information but also enhances prediction accuracy by reducing feature redundancy and model complexity.

**The main contributions of this work are summarized as follows:**

- **Development of a practical framework** for optimizing filter replacement scheduling in gas separation systems based on CFRP membranes.
- **Integration of spatial and temporal information** using advanced CNN and xLSTM architectures for more accurate fatigue prediction in CFRP membranes.
- **Feature optimization** via the Mutual Information algorithm to improve prediction accuracy and reduce computational complexity.
- **Application of ensemble learning classifiers** to enhance stability and robustness of the final predictions.

## 2. Related works

In recent years, numerous studies have investigated fatigue detection and damage diagnosis in CFRP composites using sensor-based SHM techniques. These works employ a variety of feature extraction methods and learning algorithms; however, differences in robustness, generalization capability, and spatiotemporal modeling remain evident across existing approaches. Some of recently researches have been investigated in this section.

Yoshimori et al. [13] proposed a multi-timescale fatigue damage prediction framework for carbon fiber composites using machine learning techniques such as Gaussian Process Regression (GPR) and Support Vector Machines (SVM). Their approach models the effects of cyclic loading conditions over time and focuses on predicting fatigue-induced damage mechanisms, specifically fiber breakage and matrix cracking. Although the data split for training and testing was not explicitly stated, the authors employed cross-validation techniques to evaluate model performance. Their study demonstrated the potential of combining physics-based time-scale analysis with data-driven methods.

Post et al. [14] investigated the use of machine learning for identifying the onset of damage in CFRP composites. They applied Random Forest and Gradient Boosting Machines to classify damage initiation using strain and displacement data collected under both quasi-static and fatigue loading scenarios. The main damage types considered included crack initiation and fiber-matrix debonding. The dataset was divided into 70% for training and 30% for testing, and five-fold cross-validation was used to ensure model generalization. Their results highlighted the feasibility of early diagnostics using ensemble-based classifiers.

Liu et al. [15] developed data-driven techniques to characterize delamination damage in composite materials using machine learning. Their approach combined Convolutional Neural Networks (CNNs), Support Vector Machines (SVMs), and k-Nearest Neighbors (k-NN) to analyze sensor signal data. The study specifically focused on identifying delamination using edge-based features and signal patterns. Although the data split configuration was not described in detail, the model’s effectiveness in distinguishing delaminated from healthy samples was emphasized.

Wu et al. [16] introduced a hybrid deep learning model called ALSTM-CNN for predicting the residual life of CFRP composites. The model integrates attention-based Long Short-Term Memory (LSTM) networks with CNNs to jointly capture spatial and temporal characteristics of fatigue degradation. Their dataset was divided into 80% training and 20% testing subsets. The framework was particularly suited for modeling time-series fatigue data and showed improved accuracy in estimating remaining useful life compared to traditional LSTM or CNN models alone.

Alkunte and Fidan in [17] proposed a machine learning-based approach for predicting the fatigue life of functionally graded materials fabricated via material extrusion. They used models such as XGBoost, Support Vector Regression (SVR), and Decision Trees to assess fatigue performance based on printing parameters and material properties. Although the study did not detail the data splitting strategy, it successfully demonstrated the feasibility of using regression algorithms for fatigue life estimation in 3D-printed composites.

Arnold et al. [18] presented a machine learning framework for estimating stiffness degradation and fatigue life in polymer composite laminates. They employed Random Forest and Gaussian Process Regression (GPR) models trained on handcrafted features derived from material specifications and load history. The dataset was split into 80% for training and 20% for testing. Their work demonstrated high accuracy in predicting stiffness loss and was aimed at informing structural design and durability assessments.

Gaurav in [19] utilized classical regression techniques such as Lasso, Ridge Regression, and SVR to predict the residual strength of fiber-reinforced polymer (FRP) composites subjected to fatigue. The study aimed at quantifying the degradation of mechanical properties over time, using a dataset split into 70% training and 30% testing. The model outputs were validated against experimental data, demonstrating the usefulness of simple regression models in residual property estimation.

Liu et al. [20] developed a transferable deep reinforcement learning (DRL) framework for fatigue life prognosis in composite structures. The method used a recurrent neural network backbone within a reinforcement learning setting to predict future fatigue behavior under varying loading conditions. Although the data split was not explicitly provided, the approach showed strong generalization capability across different composite configurations, addressing the challenge of domain adaptation in fatigue modeling.

Hussain et al. [21] proposed a transfer learning method based on Temporal Convolutional Networks (TCNs) for structural health monitoring of composites. By leveraging pre-trained temporal models, the method aimed to improve performance in scenarios with limited labeled data. The study focused on detecting damage progression in composite structures using time-series sensor signals. Specific details regarding data splitting were not provided, but the proposed approach achieved promising results in damage classification tasks.

Hasan et al. [22] presented a model for predicting fatigue life in reinforced membrane materials used in polymer electrolyte membrane fuel cells. The approach combined plastic energy-based degradation analysis with machine learning, though the specific type of ML model was not disclosed. The study emphasized the role of cumulative plastic energy as a damage metric for membrane degradation. While the data split was not mentioned, the method demonstrated potential for fatigue life estimation under fuel cell operating conditions.

Liu et al. [23] proposed a deep transfer learning-based approach for damage detection in composite structures by integrating monitoring data with physical mechanism models. Their method utilizes pre-trained deep learning networks to extract generalized features from vibration signals, which are then fine-tuned using data from target composite structures. By fusing data-driven models with domain knowledge from physics-based simulations, the study enhances detection accuracy even under limited training data conditions. This hybrid approach bridges the gap between purely empirical learning and physics-informed modeling, making it suitable for practical SHM applications involving complex composite behaviors.

De Carvalho Monson et al. [24] introduced a novel damage classification framework for composite materials using time-frequency analysis combined with deep learning. The approach involves transforming acoustic emission signals into time-frequency representations via the Wigner–Ville distribution (WVD), which are then fed into a Convolutional Neural Network (CNN) for classification. This method captures both temporal and spectral characteristics of structural responses, enabling accurate discrimination of various damage modes. The study demonstrates that WVD-based spectrograms provide richer signal representations compared to traditional Fourier-based methods, resulting in improved classification performance.

Guo et al. [25] developed an adversarial transformer-based model for detecting anomalous patterns in Lamb wave signals used in structural health monitoring. The proposed architecture leverages transformer encoders with adversarial training to enhance feature robustness and sensitivity to subtle signal changes associated with early-stage damage. The model effectively distinguishes between normal and anomalous wave patterns in noisy environments, demonstrating superior performance over conventional CNN and RNN-based methods. This work highlights the potential of transformer architectures in guided-wave-based SHM, particularly when combined with adversarial learning for improved generalization.

Despite the significant progress achieved by recent machine learning and deep learning approaches for CFRP fatigue diagnostics, several critical limitations remain insufficiently addressed. One major challenge lies in the robust handling of noisy sensor data, which is inherent in real-world SHM systems due to environmental disturbances, sensor aging, and operational variability. Many existing deep learning models assume relatively clean or preprocessed inputs, making them vulnerable to performance degradation under realistic noise conditions.

Another important limitation concerns the generalization capability of current models to unseen loading conditions or operational scenarios. Most reported studies evaluate performance under fixed or narrowly defined fatigue loading profiles, which restricts their applicability to real industrial environments where loading patterns, boundary conditions, and operational stresses may vary significantly over time.

Furthermore, although CNN-based models have demonstrated strong performance in extracting localized signal features and LSTM-based architectures are effective in modeling temporal dependencies, their combined use in a unified spatiotemporal framework for CFRP fatigue diagnostics remains relatively limited. Existing studies often treat spatial and temporal characteristics independently or rely on single-stream architectures, which may fail to capture the complex interaction between localized damage mechanisms and long-term fatigue evolution.

In addition, many deep learning-based SHM approaches focus primarily on end-to-end accuracy improvements without explicitly addressing feature redundancy, model interpretability, or robustness, which are crucial for practical deployment in safety-critical systems. These gaps highlight the need for hybrid architectures that integrate spatial and temporal learning, incorporate feature optimization strategies, and enhance generalization under noisy and variable operating conditions [31].

Motivated by these limitations, the present study proposes a hybrid CNN–xLSTM framework that explicitly addresses the aforementioned gaps. By combining spatial feature extraction from raw sensor signals with long-range temporal modeling, the proposed approach captures both localized fatigue-induced signal distortions and their progressive evolution. Moreover, the integration of Mutual Information-based feature selection and ensemble learning improves robustness to noise, reduces feature redundancy, and enhances generalization performance, making the framework more suitable for real-world SHM applications involving CFRP structures.

## 3. Proposed method

In this section, the proposed methodology for predicting fatigue in CFRP membranes is discussed fully. The proposed framework introduces a novel spatiotemporal deep learning pipeline specifically designed for fatigue diagnostics in CFRP-based gas separation membranes. Unlike conventional approaches that often rely solely on handcrafted features or isolated temporal or spatial models, our method combines one-dimensional Convolutional Neural Networks (1D-CNN) for spatial feature extraction with extended Long Short-Term Memory (xLSTM) networks to capture long-range temporal dependencies in the sensor signals. The proposed framework follows a sequential spatiotemporal learning strategy. Raw one-dimensional sensor signals acquired from CFRP specimens are first processed by a CNN to extract local spatial patterns embedded in the signal waveform. The CNN output, representing high-level spatial degradation descriptors, is then reformatted as a temporal feature sequence and fed into an xLSTM network to model the temporal evolution of fatigue damage. Finally, the extracted spatial and temporal features are fused, refined through feature selection, and classified using an ensemble learning strategy.

**Fig 1:**
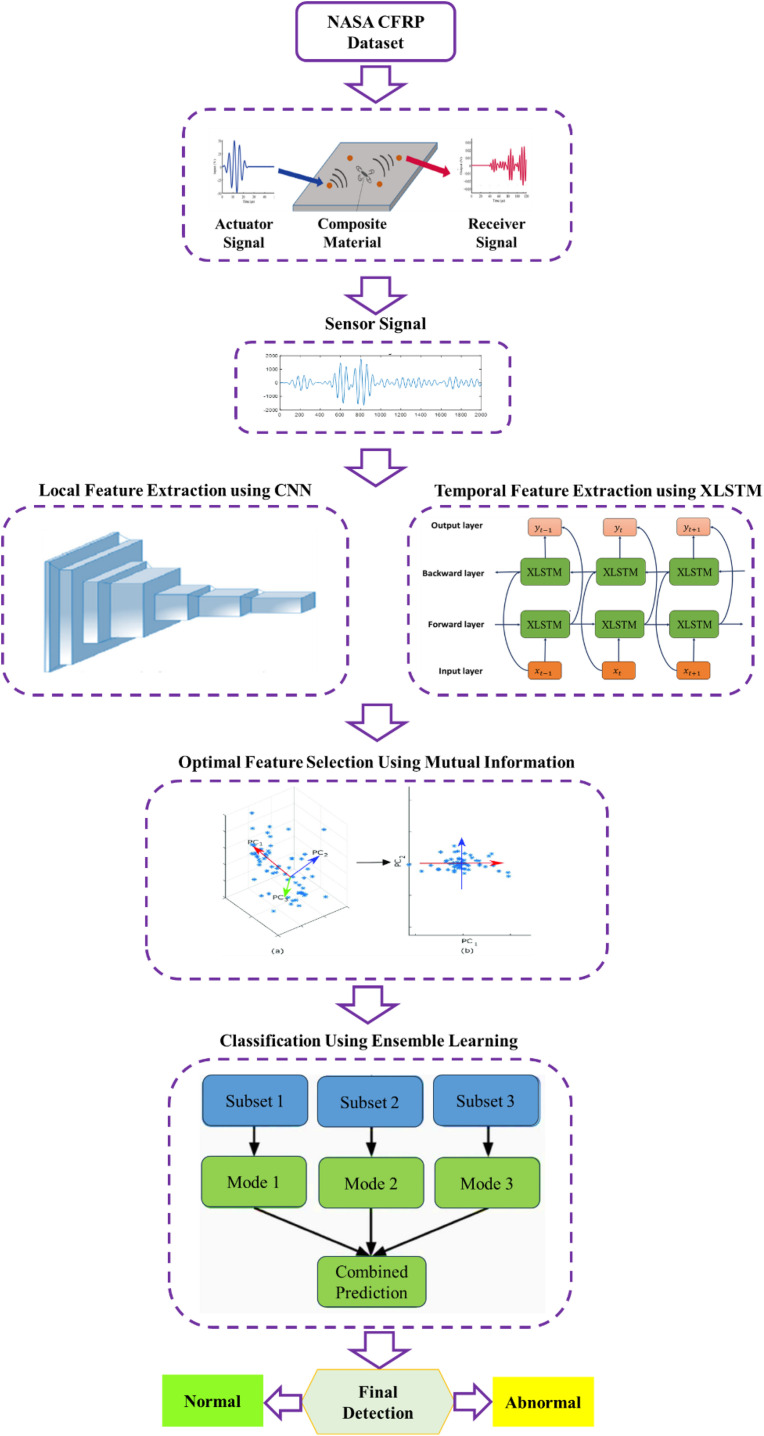
Diagram of proposed method.

Compared to prior methods, our approach provides a more comprehensive and data-efficient solution to the fatigue prediction problem, especially in scenarios with limited labeled data and high noise levels typical of real-world composite monitoring systems. The diagram of the proposed method is shown in Fig 1.

### 3.1. Spatial feature extraction using Convolutional Neural Networks (CNN)

In the proposed framework, the input to the 1D-CNN consists of raw time-domain sensor signals collected from CFRP specimens during cyclic loading experiments. Each signal is represented as a one-dimensional vector of constant length, corresponding to guided ultrasonic wave responses recorded by PZT sensors.

In the context of one-dimensional sensor signals, spatial features refer to localized patterns within the signal waveform, such as abrupt amplitude changes, wave packet distortions, and local frequency variations, which are strongly associated with fatigue-induced material degradation.

Convolutional Neural Networks (CNNs) are used to automatically learn spatial patterns such as localized degradation, crack propagation, or material microstructure changes.

Suppose the input data $𝐗\in R^{n\times m}\;$ represents n samples and m features per sample (e.g., stress, strain, cycle number, etc.). Each convolutional layer applies a filter 𝐖 to local regions of 𝐗.

A 1D convolution at position i can be defined as [26]:


$$s(i)=(W*X)(i)=\sum_{j=0}^{k-1}W(j).X(i+j)$$ (1)

where:

• k is the kernel size (e.g., k=3),

• * denotes the convolution operator,

• s(i) is the output at position i.

The output is then passed through a nonlinear activation function σ, commonly ReLU:


a(i)=σ(s(i))=max(0,s(i)) (2)

Pooling layers such as MaxPooling or AveragePooling are applied to reduce the spatial dimensionality:


$$p(i)=\underset{j\in \text{pool}\_\text{region}}{\text{max}}a(j)$$ (3)

The 1D-CNN processes these signals directly without handcrafted feature extraction. Convolutional filters slide along the temporal axis of the signal to capture localized waveform characteristics such as amplitude variations, signal distortions, and frequency-related patterns, which are indicative of microstructural fatigue damage. These learned representations are referred to as spatial features, as they encode local structural characteristics embedded within the signal shape.

Thus, CNN extracts multi-scale spatial features while preserving essential degradation information. Multiple convolutional blocks can be stacked to capture both low-level and high-level spatial features.

### 3.2. Temporal feature extraction using extended LSTM (xLSTM)

The output of the final convolutional layer is a feature map with dimensions T×F, where T denotes the reduced temporal resolution and F represents the number of learned spatial feature channels. This feature map is reshaped into a sequential format and used as the input to the xLSTM network.

The xLSTM processes this sequence to capture long-range temporal dependencies and progressive fatigue trends across consecutive loading cycles. This design allows the model to jointly analyze instantaneous structural patterns and their temporal evolution.

Long Short-Term Memory (LSTM) networks are specialized Recurrent Neural Networks (RNNs) designed to capture long-term dependencies in sequences. Fatigue phenomena evolve over time, making LSTM an ideal tool.

The xLSTM network enhances the traditional LSTM by deeper stacking, attention mechanisms, or residual connections [27].

At each time step t, given the input vector $X_{t}$ and the previous hidden state $h_{t-1}$, the LSTM computes:

1 Forget gate:

$$f_{t}=\sigma ({𝐖}_{fX_{t}}+{𝐔}_{fh_{t-1}}+b_{f})$$ (4)

2 Input gate:

$$i_{t}=\sigma ({𝐖}_{iX_{t}}+{𝐔}_{ih_{t-1}}+b_{i})$$ (5)

3 Candidate cell state:

$${\overset{\sim }{C}}_{t}=\text{tanh}({𝐖}_{cX_{t}}+{𝐔}_{ch_{t-1}}+b_{c})$$ (6)

4 Cell state update:

$$C_{t}=f_{t}⊙C_{t-1}+i_{t}⊙{\overset{\sim }{C}}_{t}$$ (7)

5 Output gate:

$$o_{t}=\sigma \left({𝐖}_{oX_{t}}+{𝐔}_{oh_{t-1}}+b_{o}\right)$$ (8)

6 Hidden state:

$$h_{t}=o_{t}⊙\text{tanh}(C_{t})$$ (9)

where:

• σ is the sigmoid activation,

• ⊙ is the element-wise multiplication.

The xLSTM architecture in this research includes attention mechanisms in which an attention weight $\alpha_{t}$ is assigned to each hidden state [27]:


$$\alpha_{t}=\frac{\text{exp}(e_{t})}{\sum_{k=1}^{T}\text{exp}(e_{k})},\;\;e_{t}=v^{T}\;\text{tanh}(Wh_{t}+b)$$ (10)

Thus, important time steps (representing critical fatigue events) are weighted more heavily.

### 3.3. Feature fusion

After spatial and temporal feature extraction, feature fusion is performed by concatenating the high-level spatial descriptors obtained from the CNN with the temporal representations learned by the xLSTM. This fusion operation produces a unified spatiotemporal feature vector that captures both localized damage characteristics and their progression over time. The fused features are subsequently passed to the Mutual Information-based feature selection stage to retain the most informative attributes for fatigue state classification.

Let:

•${𝐅}_{\text{spatial}}\in R^{n\times d_{s}}$ be the CNN output (spatial features),

•${𝐅}_{\text{temporal}}\in R^{n\times d_{t}}\;$ be the xLSTM output (temporal features).

The fusion operation is typically a simple concatenation:


$${𝐅}_{\text{combined}}=[{𝐅}_{\text{spatial}},{𝐅}_{\text{temporal}}]\in R^{n\times (d_{s}+d_{t})}$$ (11)

This step ensures that both spatial structure and temporal evolution are jointly considered in downstream tasks.

### 3.4. Optimal feature selection using Mutual Information (MI)

After fusion, many redundant or irrelevant features may exist. To retain only the informative ones, Mutual Information (MI) is computed between each feature and the target label **Y** (fatigue/safe) [28].

MI between a feature **X** and label **Y** is:


$$\begin{array}{c} I(X;Y)=\sum_{x\in X}\sum_{y\in Y}p(x,y)\text{log}\left(\frac{p(x,y)}{p(x)p(y)}\right) \end{array}$$ (12)

where:

• p(x,y) is the joint probability distribution,

• p(x) and p(y) are the marginals.

The steps for selecting optimal features by the MI algorithm in this research are as follows:

1. Estimate p(x,y), p(x), and p(y) from data (e.g., using histograms, kernel density estimation).
2. Calculate $I(X_{j};Y)$for each feature$X_{j}$.
3. Rank features by MI scores.
4. Select the top-k features with the highest MI scores.

This reduces the feature space from $d_{f}$ to $d_{selected}$ $(d_{selected}\ll d_{f}$). Thus, the retained features are those most informative for predicting fatigue behavior.

### 3.5. Classification using ensemble learning

In this study, Bagging ensemble learning was employed using Decision Trees as the base classifiers. In this case, we can say that our ensemble learning algorithm is random forest.

The ensemble learning algorithm based on decision trees for classification works by training several decision trees as base models, and then combining their predictions to obtain the final prediction. Here are three main formulas for this algorithm [29]:

a) **Final Prediction (Majority Voting):**

The final prediction is obtained through majority voting from the predictions made by the different trees:


$$\hat{y}=\text{mode}\left(h_{1}(x),h_{2}(x),\ldots ,h_{M}(x)\right)$$ (13)

where $h_{i}(x)$ is the prediction made by the i-th decision tree, and $\hat{y}$ is the final prediction.

b) **Gini Impurity (Split Criterion for Decision Tree):**

Decision trees typically use criteria like Gini Impurity to split the data. This measure for a node is calculated as:


$$\begin{array}{c} Gini(t)=1-\sum_{i=1}^{C}p_{i}^{2} \end{array}$$ (14)

where $p_{i}$ is the probability of samples belonging to class i, and C is the number of classes.

c) **Overall Error of Base Models (Total Error):**

Suppose $h_{i(x)}$ is the prediction of model i. The overall error of the ensemble model is calculated as the average error of the base models:


$$\begin{array}{c} {\text{Error}}_{\text{ensemble}}=\frac{1}{M}\sum_{i=1}^{M}\text{Error}\left(h_{i}(x),y\right) \end{array}$$ (15)

where $\text{Error}(h_{i}(x),y)$ is the error of model $h_{i(x)}$ for sample x, and M is the number of base models (decision trees).

The proposed methodology offers a comprehensive solution for fatigue prediction in CFRP membranes. The CNN efficiently captures local spatial patterns indicative of early fatigue, while the xLSTM models the long-term progression of material degradation. The fusion of spatial and temporal features ensures a holistic representation of the system state. By applying Mutual Information-based feature selection, the method eliminates redundancy, retains only the most significant information, and accelerates learning. Finally, ensemble learning ensures robust classification performance even in the presence of noisy or limited data. Together, these components create a scalable, interpretable, and highly accurate fatigue prediction framework capable of determining optimal filter replacement times with minimal error and maximal reliability.

## 4. Results and discussions

In this section, the performance of the proposed fatigue prediction method was evaluated using the NASA-CFRP dataset. All simulations and model training were conducted in MATLAB 2023, leveraging its deep learning and statistical analysis capabilities. Key evaluation metrics, including accuracy, precision, recall, and F1-score, were used to assess the model’s effectiveness and compare it against existing approaches.

### 4.1. Database

The NASA-CFRP dataset is a publicly available collection developed by the National Aeronautics and Space Administration (NASA) to support research in fatigue prediction and structural health monitoring of carbon fiber reinforced polymer (CFRP) materials [30]. This dataset includes time-series data such as strain and load collected from CFRP composite specimens subjected to cyclic loading conditions. It provides detailed information on material degradation over time, making it highly valuable for the development and evaluation of machine learning and deep learning models aimed at predicting fatigue life and detecting early signs of failure.

The NASA-CFRP dataset comprises fatigue sensor signals obtained from carbon/epoxy composite specimens with a [0/45/90/–45]s quasi-isotropic layup configuration. This stacking sequence is designed to provide multidirectional strength and reflects practical aerospace-grade laminate structures.

**Fig 2:**
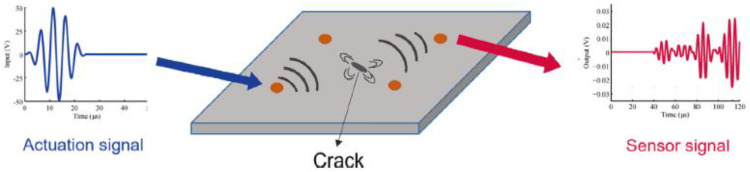
Guided ultrasonic wave propagation with PZT actuator and receiver [23].

In this database, Piezoelectric sensors (PZT) act as both actuators and sensors and are mounted on a thin CFRP plate. In the testing process, ultrasonic guided waves are induced in the substrate material by the actuator and propagate through the material structure. The sensors then receive these waves as output signals. Any disturbances, such as reflections from internal damage in the substrate material, are detected by the signals recorded from the sensors. In Fig 2, a schematic of the Lamb wave propagation and recording system by PZT sensors for fatigue detection in composite materials is presented.

In the NASA-CFRP dataset, fatigue-related damage mechanisms such as microcracking, matrix degradation, and delamination directly affect the propagation behavior of PZT-based guided ultrasonic waves. As fatigue damage accumulates, these defects introduce scattering, attenuation, and waveform distortion, leading to measurable changes in signal amplitude, phase, and time-of-flight.

The signals are acquired under cyclic mechanical loading conditions, where accumulated fatigue inherently influences signal characteristics. Although temperature is controlled during the experiments, minor variability may arise from sensor coupling conditions and measurement noise. These factors reflect realistic SHM operating environments and motivate the use of robust feature extraction and classification techniques.

### 4.2. Evaluation criteria

The main metrics used for evaluating the proposed approach in this study are classification accuracy, precision, recall, and F1 score. Classification accuracy is the primary metric in classification problems and is determined by the ratio of correct predictions (both true positives and true negatives) to the total number of predictions. The following metrics are utilized to assess the performance of the proposed method.

In follow equations, TP refers to true positives (correctly predicted fatigued polymers), TN refers to true negatives (correctly predicted healthy polymers), FP represents false positives (incorrectly predicted fatigued polymers), and FN represents false negatives (incorrectly predicted healthy polymers).


$$Accuracy(acc)=\frac{TP+TN}{TP+TN+FP+FN}$$ (16)

$$precision=\frac{TP}{TP+FP}$$ (17)

$$recall=\frac{TP}{TP+FN}$$ (18)

$$F1score=2*\frac{recall*precision}{recall+precision}$$ (19)

### 4.3. Simulation setting

In this study, the dataset consists of 252 sensor signals from healthy CFRP materials and 252 from damaged CFRP materials, leading to a total of 504 signals. To address the limited size of the original dataset and improve model generalization, signal-level data augmentation techniques were employed. Specifically, three methods were applied: (1) Gaussian noise injection (mean = 0, std = 0.05), (2) random time shifting within ±50 time steps, and (3) amplitude scaling with a factor randomly selected from [0.9, 1.1].

Gaussian noise injection was employed with a zero mean and a standard deviation of 0.05. This value was determined empirically through preliminary sensitivity analysis to ensure that the injected noise magnitude remains significantly lower than the dominant signal amplitude. As a result, the augmentation simulates realistic sensor noise arising from environmental disturbances, electronic interference, and measurement uncertainty, without altering fatigue-related waveform structures.

Time-shifting augmentation was applied within a range of ±50 time steps, which corresponds to minor temporal misalignments commonly observed in guided-wave propagation due to operational variability or sensor triggering uncertainty. This operation preserves the underlying waveform shape and fatigue-induced distortions, thereby maintaining the integrity of damage-related patterns.

In addition, amplitude scaling was limited to a narrow range of [0.9, 1.1] to reflect plausible variations in excitation energy and sensor coupling conditions. Importantly, none of the applied augmentation techniques modify the sequential order or progressive evolution of the signal, ensuring that fatigue crack propagation patterns and temporal degradation trends remain physically consistent.

After applying data augmentation, the number of signals increased to 3024, with each signal having a length of 2000. Feature extraction was performed using two distinct networks: a CNN, which extracted 400 spatial features, and an xLSTM network, which extracted an additional 400 temporal features. The specifications of CNN and xLSTM networks are presented in Table 1. The total number of features extracted was thus 800, which were subsequently reduced to 200 using Mutual Information (MI) feature selection. The number of selected features after Mutual Information (MI)-based ranking was determined through a trade-off analysis between classification performance and model complexity. Specifically, preliminary experiments were conducted by gradually increasing the number of retained features and evaluating the corresponding classification accuracy on a validation subset. The results indicated that performance improvements saturated beyond approximately 200 features, while further increasing the feature count led to marginal gains at the cost of higher computational complexity and increased risk of overfitting. Therefore, selecting the top 200 features represents a balanced choice that preserves the most informative spatiotemporal characteristics while maintaining computational efficiency and model robustness. This threshold corresponds to retaining 25% of the original fused feature set and is consistent with common practices in feature selection for high-dimensional SHM data.

**Table 1:** The specifications of proposed CNN and xLSTM networks.

	Number of Layers	Filter Size	Number of Features Extracted	Activation Function	Number of Neurons (xLSTM Units)	Learning Rate	Optimizer	Epochs	Batch Size
CNN	5	1*3	400	ReLU	N/A	0.001	Adam	50	32
xLSTM	3	N/A	400	Tanh	128	0.001	Adam	50	32

The dataset was divided into training and testing sets in a 70:30 ratio, resulting in 2117 training samples and 907 testing samples. we have ensured that signals from a physical composite sample do not appear in both the training and test sets. This prevents potential data leakage and better assesses the generalization ability of the model. All simulations were conducted using MATLAB 2023.

### 4.4. Evaluation of results

To evaluate the performance of the proposed method for fatigue prediction in carbon fiber reinforced polymers (CFRPs) used in gas separation polymeric membranes, a series of experiments were conducted using the NASA-CFRP dataset. The main objective of this evaluation is to assess the classification accuracy, model convergence, and error reduction during training [32]. The proposed approach involves spatial feature extraction using a CNN, temporal feature extraction via an xLSTM network, feature fusion, optimal feature selection using Mutual Information, and final classification through ensemble learning.

**Fig 3:**
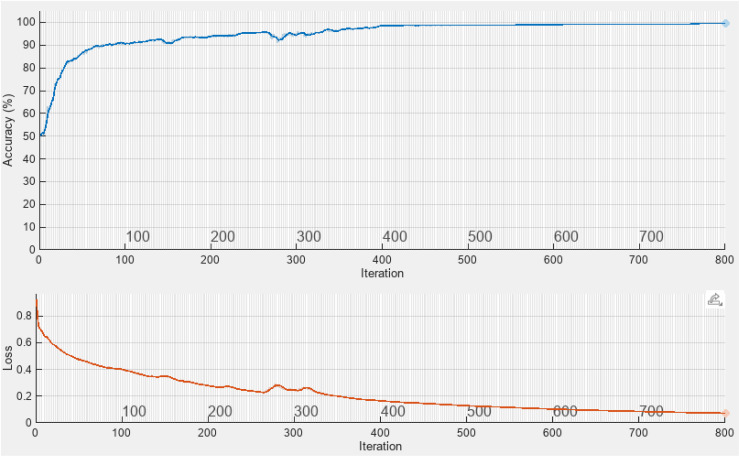
Convergence curve of Proposed CNN Network.

The top plot in the Fig 3 shows the CNN model’s accuracy over training iterations, while the bottom plot depicts the loss reduction during the same process. Initially, the model accuracy starts around 50% and rapidly increases, surpassing 90% within the first 100 iterations. The accuracy then gradually improves, stabilizing between 99% and 100% toward the end of training, which indicates successful convergence and absence of overfitting at this stage.

In the loss plot, the loss value starts above 0.8 and continuously decreases throughout the training, eventually dropping below 0.05. This consistent decline in loss reflects effective learning of fatigue-related patterns from the input data. The minor fluctuations observed mid-training are typical and followed by steady stabilization, confirming that the model has been trained adequately.

**Fig 4:**
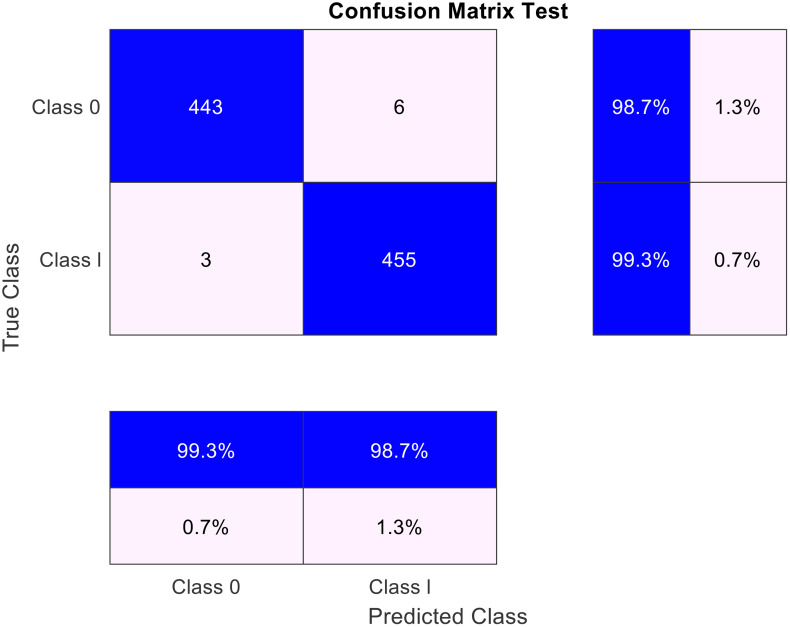
Confusion Matrix on the test data.

The results of the confusion matrix for the test data in Fig 4 demonstrate the strong performance of the proposed model in classifying both classes. For class 0 (healthy), the model correctly predicted 443 samples (sensitivity 98.7%) and misclassified only 6 samples as class 1 (defective) (error rate 1.3%). In contrast, for class 1 (defective), the model correctly identified 455 samples (sensitivity 99.3%) and misclassified only 3 samples as class 0 (error rate 0.7%). The overall accuracy of the model for both classes is above 98%, indicating a high level of reliability in detecting the condition of CFRP composites. These results confirm the effectiveness of the combined CNN-xLSTM method and feature selection based on Mutual Information in distinguishing between healthy and defective samples.

**Fig 5:**
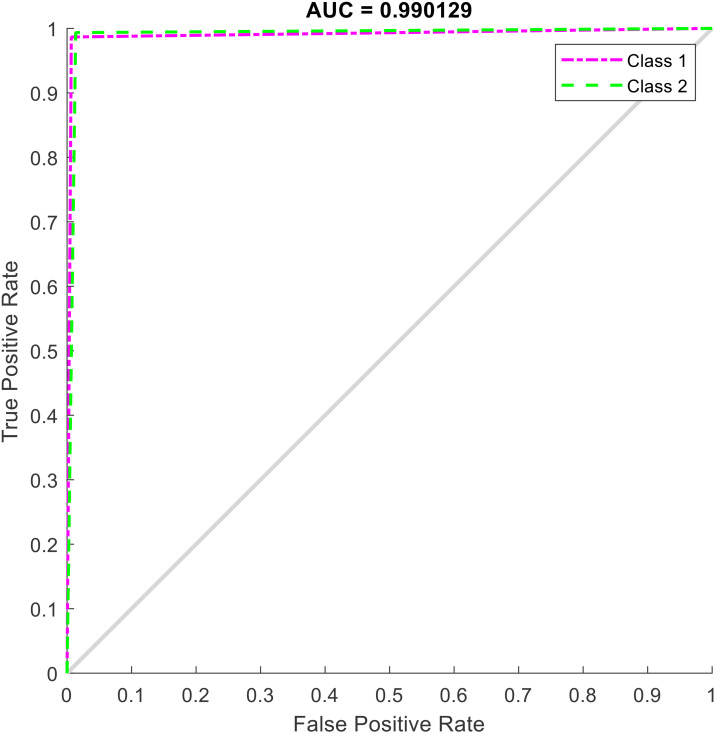
ROC Curve on the test data.

The results of the ROC curve with an AUC (Area Under the Curve) value of 0.990129, shown in Fig 5, indicate the exceptional performance of the proposed model in distinguishing between the healthy and defective classes. The AUC value close to 1 (the maximum possible) confirms that the model has an extremely high ability to correctly classify positive (defective) and negative (healthy) samples. This result, which is consistent with the previous confusion matrix and is accompanied by high sensitivity (True Positive Rate) and specificity (1 – False Positive Rate), demonstrates that the combined CNN-xLSTM model not only performs well in classification but also in ranking the probability of samples belonging to each class. Such a level of accuracy makes the model a reliable option for practical applications in monitoring the health of CFRP structures.

**Fig 6:**
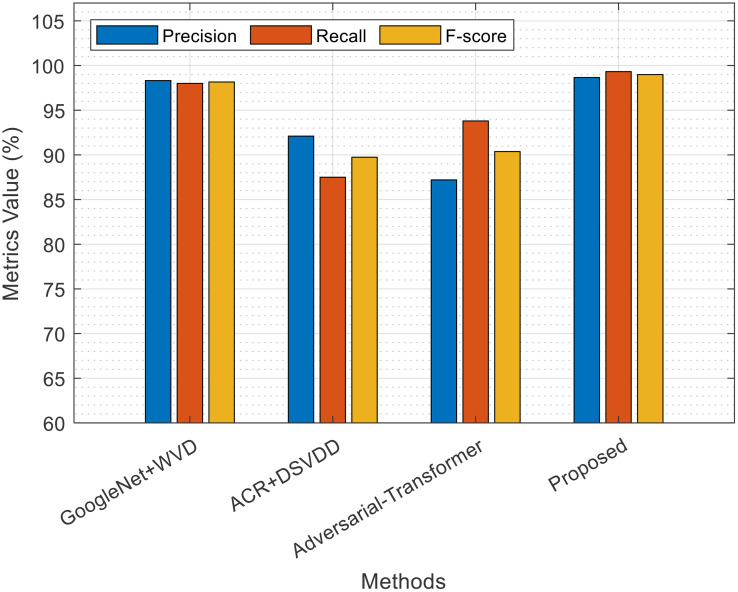
Comparison of results in terms of evaluation criteria.

The comparative results of the proposed method with other existing methods (GoogleNet+NVID, ACR+DSVDD, and Adversarial+Transformer) shown in Fig 6, based on the metrics of Precision, Recall, and F-Score1, indicate the clear superiority of the proposed method. According to the provided values, the combined CNN-xLSTM method outperforms the comparative methods in all evaluation metrics, including Precision, Recall, and F1 Score. This superiority is especially evident in the F1 Score, which is a combination of Precision and Recall, and highlights the model’s well-balanced performance in simultaneously detecting both positive and negative samples. Such results confirm the effectiveness of the proposed architecture in extracting spatio-temporal features and optimizing feature selection for fatigue classification in CFRP composites. A comparison of these results with Transformer-based methods and adversarial approaches also shows that, despite its relative simplicity, the proposed method offers higher generalizability and accuracy.

The comparative results between the proposed method (CNN + xLSTM+Ensemble Learning) and other existing methods in Table 2 show that the proposed method achieves better performance with an accuracy of 99.00%. Methods based on different architectures, such as GoogleNet + WVD (98.16%), EfficientNet b0 + WVD (97.03%), Xception+WVD (97.77%), and ResNet50 + WVD (98.52%), although achieving high accuracy, still perform lower than the proposed method. Additionally, methods like ACR+DSVDD (95.1%) and Adversarial Transformer (95.2%), clearly show weaker performance compared to the proposed method.

**Table 2:** Comparison of results in terms of Accuracy.

Method	Ref	Year	Accuracy
GoogleNet+ WVD	[24]	2024	98.16
EfficientNet b0+ WVD	[24]	2024	97.03
Xception+ WVD	[24]	2024	97.77
ResNet 50+ WVD	[24]	2024	98.52
ACR+DSVDD	[25]	2025	95.1
Adversarial Transformer	[25]	2025	95.2
CNN+XLSTM+MI+Ensemble Learning	-	-	99.00

Regarding transformer-based models, their comparatively lower performance can be attributed to the characteristics of the NASA-CFRP dataset. Transformer architectures typically require large-scale datasets to fully exploit self-attention mechanisms and long-range dependency modeling. However, the limited number of labeled fatigue samples, class imbalance, and sensor noise in the dataset restrict their learning capacity and increase susceptibility to overfitting. In contrast, the proposed CNN–xLSTM architecture benefits from localized convolutional feature extraction and gated temporal modeling, which are better suited to capturing fatigue-induced signal variations in data-constrained SHM scenarios.

Although the achieved classification accuracy exceeds 99%, several measures were explicitly adopted to mitigate the risk of overfitting given the limited size of the original dataset. First, a strict sample-wise separation was enforced between training and testing sets to prevent data leakage. Second, signal-level data augmentation was applied to increase sample diversity while preserving physical realism. Third, Mutual Information-based feature selection was employed to reduce dimensionality and eliminate redundant features.

In addition, ensemble learning via a Bagging-based classifier was used to improve generalization and reduce variance, particularly in small-sample scenarios. Regularization mechanisms, including dropout and early stopping, were also incorporated during network training. The consistently high performance across multiple evaluation metrics, rather than accuracy alone, indicates that the proposed model learns meaningful fatigue-related patterns rather than memorizing the training data.

This superiority of the proposed method can be attributed to the intelligent combination of spatial feature extraction by CNN, modeling of temporal dependencies with xLSTM, and the use of ensemble learning to enhance the reliability of predictions. The results of this comparison clearly demonstrate that the proposed approach outperforms not only older methods but also the latest Transformer-based approaches.

The combined CNN-xLSTM approach operates with three key advantages over existing methods: 1) Intelligent integration of spatial-temporal features that enables the simultaneous identification of localized damage and gradual degradation trends, 2) Targeted dimensionality reduction with Mutual Information, which enhances the model’s focus on key fatigue indicators by eliminating noise and redundant features, and 3) An ensemble learning framework that ensures model reliability in real-world operational conditions by reducing prediction variance. These three components together have achieved an accuracy of 99%, surpassing competitors by at least 0.5%.

### 4.5. Ablation study

To validate the contribution of each component in the proposed hybrid architecture, an ablation study was conducted. The objective of this analysis is to quantitatively assess the effectiveness of individual model blocks and to justify the necessity of integrating the 1D-CNN and xLSTM networks within the proposed framework. Table 3 summarizes the classification performance of these configurations in terms of accuracy and F-score.

**Table 3:** Contribution of each block in the proposed model.

Method	Accuracy	F-Score
1D CNN + Ensemble Learning	98.09	98.00
xLSTM + Ensemble Learning	97.88	97.82
CNN + xLSTM+ Ensemble Learning	98.76	98.69
CNN + xLSTM+ MI+ Ensemble Learning	99.00	98.81

The results demonstrate that using 1D-CNN alone achieves an accuracy of 98.09%, indicating that spatial patterns embedded in the signal waveform contain significant fatigue-related information. Similarly, the xLSTM-only model attains an accuracy of 97.88%, confirming the importance of modeling long-term temporal dependencies in fatigue evolution. However, both single-stream architectures show limitations when used independently. When spatial and temporal learning are combined (CNN + xLSTM), the accuracy increases to 98.76%, highlighting the complementary nature of the two feature representations. This improvement confirms that fatigue-related degradation in CFRP structures is characterized by both localized signal distortions and progressive temporal trends. Finally, incorporating Mutual Information-based feature selection further enhances performance, achieving the highest accuracy of 99.00%. This indicates that eliminating redundant and irrelevant features strengthens the discriminative capability of the fused feature set and improves generalization. Overall, the ablation study clearly demonstrates that each component of the proposed architecture plays a critical role in achieving optimal fatigue classification performance, thereby validating the effectiveness of the hybrid CNN–xLSTM framework.

## 5. Conclusion

In this study, a new approach based on deep learning for fatigue prediction in Carbon Fiber Reinforced Polymer (CFRP) membranes employed in gas separation was proposed. The integration of Convolutional Neural Networks (CNN) for spatial feature extraction with extended Long Short-Term Memory (xLSTM) networks for modeling temporal dependencies made possible a complete analysis of fatigue progression. Using the Mutual Information approach as a tool for feature selection and ensemble learning as the strategy for classification, the efficiency of the proposed method indicated excellent performance with an average prediction rate reaching 99% on the NASA-CFRP dataset. This remarkable accuracy also increases the reliability of fatigue monitoring in CFRP-based systems, and offers an instrument to help optimize the maintenance schedules and prevent loss-making failures. The results show that the presented framework considerably outperforms other methods and provides a strong and viable approach to the instantaneous fatigue prediction in advanced composite materials. Further work may study the implementation of additional sensory data and the adaptation of the method to other material systems to enhance its applicability to other industrial environments.
